# Effects of pulsing of light on the dentinogenesis of dental pulp stem cells *in vitro*

**DOI:** 10.1038/s41598-018-19395-x

**Published:** 2018-02-01

**Authors:** Hong Bae Kim, Ku Youn Baik, Hoon Seonwoo, Kyoung-Je Jang, Myung Chul Lee, Pill-Hoon Choung, Jong Hoon Chung

**Affiliations:** 1 Department of Biosystems & Biomaterials Science and Engineering, Seoul National University, Seoul, 08826 Republic of Korea; 2 Electrical and Biological Physics, Kwangwoon University, Seoul, 01897 Republic of Korea; 3 Department of Industrial Machinery Engineering, Sunchon National University, Jeollanam-do, 57922 Republic of Korea; 4 Department of Oral and Maxillofacial Surgery and Dental Research Institute, School of Dentistry, Seoul National University, Seoul, 03080 Republic of Korea

## Abstract

Low power light (LPL) treatment has been widely used in various clinical trials, which has been known to reduce pain and inflammation and to promote wound healing. LPL was also shown to enhance differentiation of stem cells into specific lineages. However, most studies have used high power light in mW order, and there was lack of studies about the effects of very low power light in μW. In this study, we applied 810 nm LPL of 128 μW/cm^2^ energy density *in vitro*. Upon this value, continuous wave (CW) irradiation did not induce any significant changes for differentiation of human dental pulp stem cells (hDPSCs). However, the membrane hyperpolarization, alkaline phosphatase activity, and intracellular oxidative stress were largely enhanced in the pulsed wave (PW) with 30% of duty cycle and 300–3000 Hz frequencies-LPL in which LED driver work in the form of square wave. After 21 days of daily LPL treatment, Western blot revealed the dentinogenesis in this condition *in vitro*. This study demonstrates that the very low power light at 810 nm enhanced significant differentiation of hDPSCs in the PW mode and there were duty cycle dependency as well as pulsing frequency dependency in the efficiency.

## Introduction

Low power light (LPL) therapy is a therapeutic modality that is increasingly being utilized by clinicians as a treatment paradigm for acute and chronic musculoskeletal injuries, osteoarthritis, inflammation, soft tissue injuries, etc.^1–4^. The red or near-infrared (NIR) light is known to be effective, but UV is known to induce apoptosis when the power density ranges between 5 to 5000 (mW/cm^2^)^5^. In addition to the light property itself, it has been reported that the biological effects of LPL depend on how it is applied.

Though many previous studies have used continuous wave (CW) mode due to its easiness to drive, some studies have shown benefits of pulsed wave (PW)^6–15^. First of all, PW reduces the temperature increase in tissues. The quench periods (Off times) permit higher intensity light that deepen the penetration of light into tissue with the same average power^14^. If the target tissue has high scattering properties, PW mode is necessary to deliver sufficient light energy into deep inside the tissue. Since bone-like tissue is one of the highest light scattering tissues, whose center is filled with multipotent stem cells, PW mode should be used to activate internal area of bone-like tissue by LPL. Secondly, PW-LPL has been reported to be more effective in some biological events^15^. These periodic light stimuli may accelerate or retard some biological processes such as the kinetics of ion channels or redox-linked proton pumps whose time scales are in a few to a hundred milliseconds^16^. It is reported that the effective pulse frequency range of PW-PBM is between 10 and 8,000 Hz, and there are specific effective values for different cell types^9,11^.

LPL is specially considered as a prospective tool due to its regulating effects on the proliferation and differentiation of stem cells^17–25^. Human dental pulp derived stem cells (hDPSCs) have attracted attentions in tissue engineering due to their multipotency in differentiation and easy acquisition from wasting teeth^26,27^. Since LPL does not remain any byproducts, the controllability of hDPSCs by LPL would make them more valuable stem cell sources^28,29^. Furthermore the functional modulation of hDPSCs *in situ* by LPL could provide solutions for reducing osteoporosis or bone regeneration. However, there have been few studies on the effects of pulsing or pulsed light on hDPSCs.

Herein, we report the effects of pulsing of LPL treatment on the differentiation of hDPSCs. To improve the clinical relevance, we used patient-derived stem cells. The duty cycle and the frequency of LPL treatment were screened based on the hDPSC differentiation. Considering the high scattering of dental tissues, sub-mW order weak LPL was examined. Additionally, the relations of intracellular reactive oxygen species (ROS) and mitochondrial activity in LPL induced hDPSC differentiation were examined following previous reports. Many reports showed that cytochrome c oxidase primarily absorbs light in mitochondrial transport chain (ETC), triggering ROS production, which have been known to be secondary signaling messengers in regulating proliferation and differentiation of stem cell^30–33^. This basic research provides information that will pave a way for efficient application of LPL for dental stem cell engineering.

## Results and Discussion

### LPL conditions for effective modulation of hDPSCs activity

**Figure 1:**
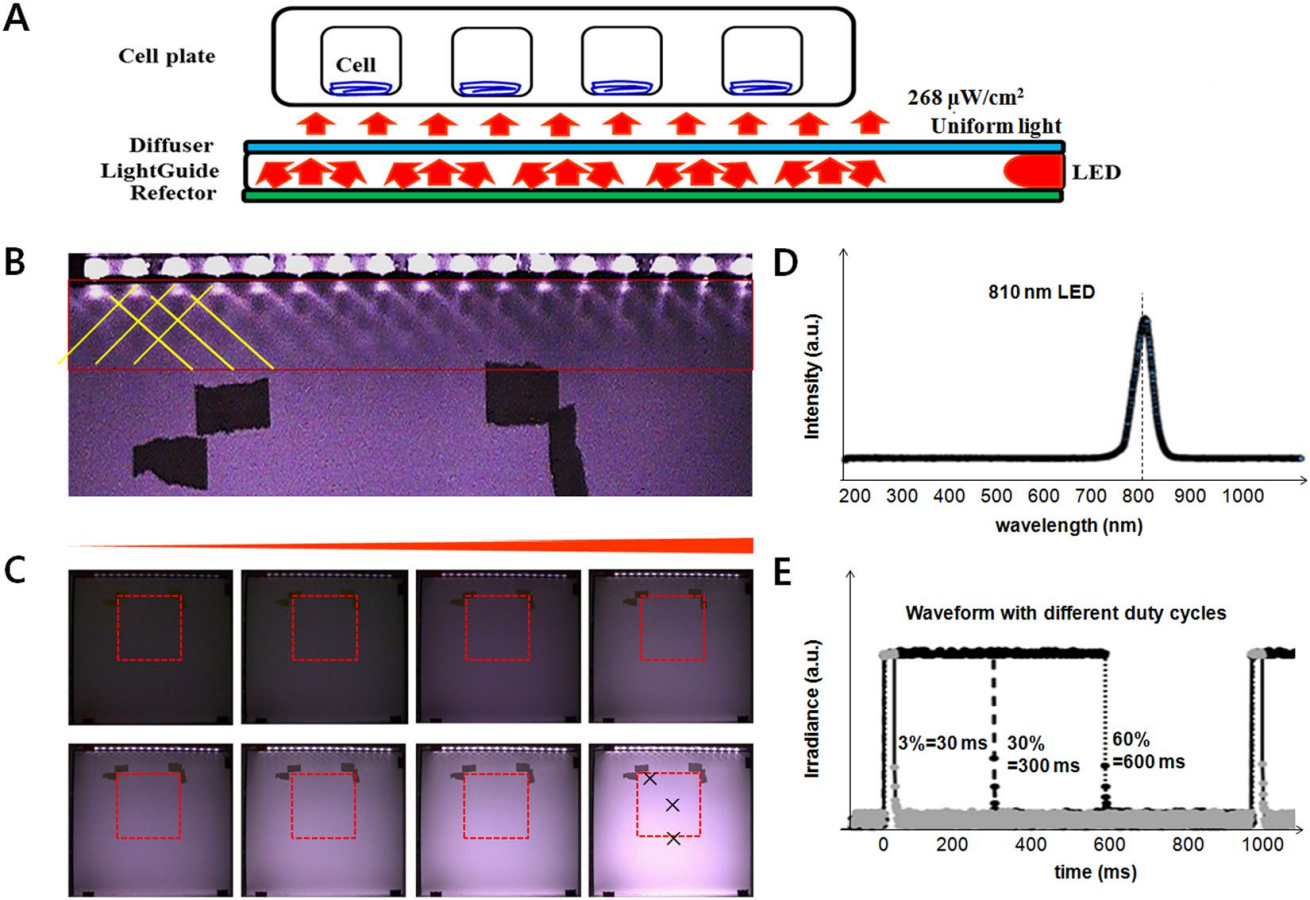
An optical device that carries light energy to objects. (**A**) A schematic of the device which is composed of LEDs as a light source, and a reflector, a light-guider and a diffuser to make the exposure light uniform. (**B**) A photo of the LED source part, from where light travels through a light-guide and becomes uniform at some distance. (**C**) Photos of the device with increasing driving voltage. Samples were put within the marked area with dotted rectangle. The variance of light intensity within this area was under 3.9%. (**D**) LEDs irradiated only near infrared 810 nm light. (**E**) Applied voltage was modulated in the form of 1 Hz square waves with different duty cycles.

Our LPL system was designed to expose cells to light through the bottom of culture dish as shown in Fig. 1A. Light from LEDs traveled through the light guide, was reflected by a reflector, diffused through a diffuser, and then reached the cell culture plates. Figure 1B shows that the light traveled through a disperser and a reflector became uniform with the variance under 3.9%. Samples were put on the area marked with dotted rectangles in Fig. 1C. We used a light emitting diode (LED) whose light was centered at 810 nm wavelength (Fig. 1D), which was known to activate cells to differentiate^28^. The applied voltage is square waveforms, so the 60% duty cycle means that LEDs were ‘on’ for 600 ms and ‘off’ for 400 ms for 1 Hz waves as shown in Fig. 1E.

**Figure 2:**
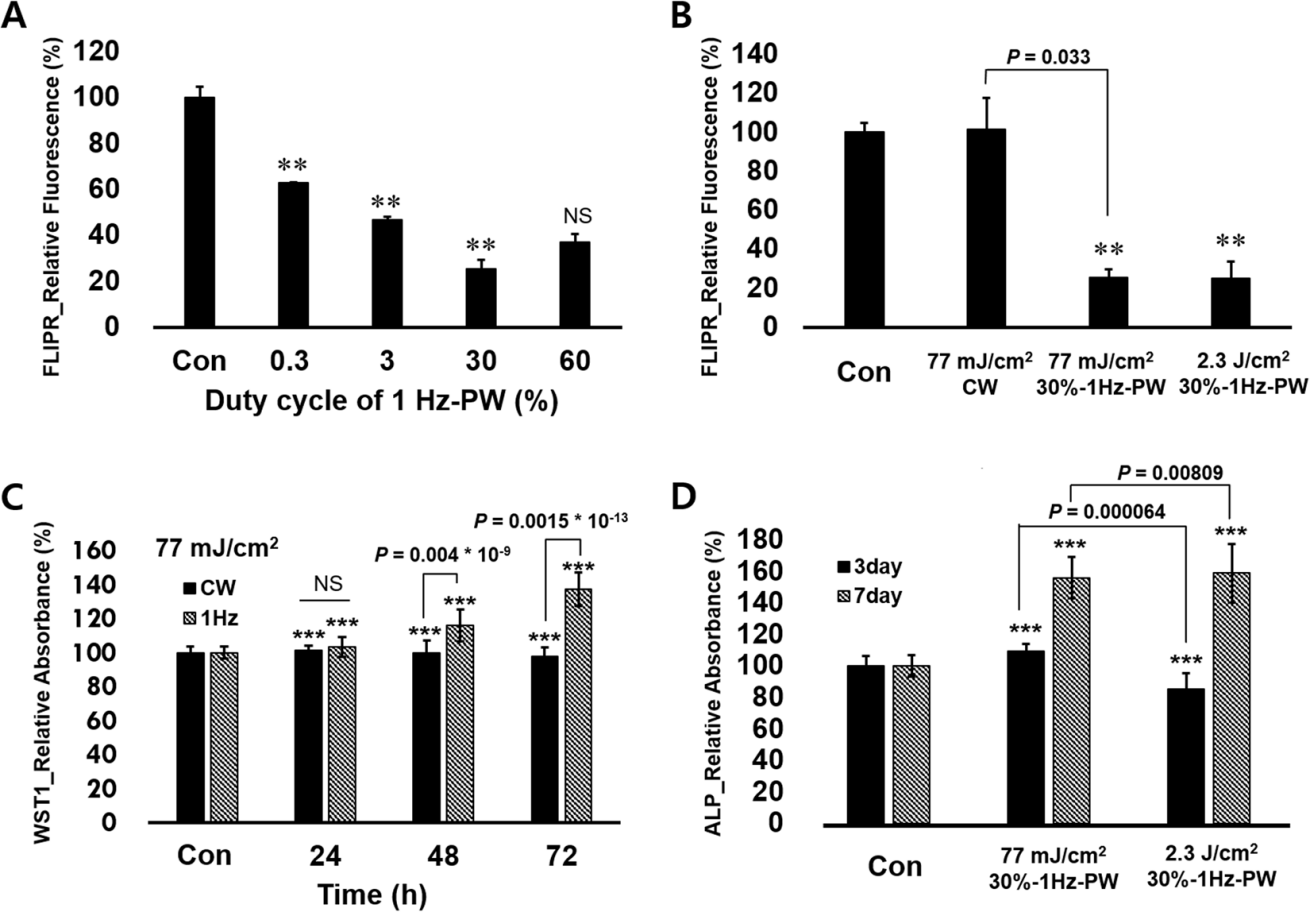
Effects of power and duty cycle of LPL on hDPSC cytoplasmic membrane potential, metabolic activity and ALP activity. (**A**) FLIPR fluorescence intensities of hDPSCs relative to control after 1 Hz PW-LPL treatment with different duty cycles 0.3 to 60% (n = 3 in each experiment and 3 replicates). (**B**) FLIPR fluorescence intensities of hDPSCs relative to control after 77 mJ/cm^2^ CW-, 77 mJ/cm^2^ PW-, or 2,310 mJ/cm^2^ PW-LPL with 30% duty cycle. The statistical analysis between CW and PW with 77 mJ/cm^2^ indicated *P* expressed 0.033 (n = 3 in each experiment and 3 replicates). (**C**) WST-1 assay absorbance relative to control after CW and 1 Hz LPL treatment with 30% duty cycle. P values between CW and PW (1 Hz) was all under 0.001 (n = 10 in each experiment and 3 replicates). (**D**) ALP activity absorbance relative to control after 77 mJ/cm^2^ and 2,310 mJ/cm^2^ 1 Hz PW-LPL applied energy density with 30% duty cycle. *P* values between 77 mJ/cm^2^ and 2.3 J/cm^2^ in PW mode was significant (n = 10 in each experiment and 3 replicates).

In order to choose LPL conditions for effective modulation of hDPSC activity on osteogenic differentiation, we firstly screened the change of cytoplasmic membrane potential (CMP). hDPSCs were subjected to 1 Hz square wave LPL with different duty cycles including 0, 0.3, 3, 30, and 60% for 10 minutes. The total energies were 0.77, 7.7, 77, and 154 mJ/cm^2^, respectively. FLIPR fluorescence reduced simultaneously with LPL treatment in all the duty cycles (Fig. 2A). When cells become hyper-polarized, the fluorescence of FLIPR dye becomes weaker. The cells were most hyperpolarized at a 30% duty cycle, even though the energy density was half comparing to 60%. In order to confirm the effects of power itself, we compared the fluorescence of four groups including control, 77 mJ/cm^2^ CW, 77 mJ/cm^2^ PW with duty cycle of 30%, and 2,310 mJ/cm^2^ PW with duty cycle of 30% (Fig. 2B). Cells underwent hyperpolarization with both PW-LPLs, however CW-LPL did not induce any changes. In addition, three times higher intensity did not induce higher polarization with same duty cycle. These results imply that the quick responses of hDPSCs to LPL do not depend on the amount of energy density, but on the discontinuity itself.

Based on the simultaneous changes in CMP, the proliferation and differentiation were also examined in long term. The cell metabolic activity was assessed at elapsed time of 1, 2, and 3 days from LPL treatments. Figure 2C shows the WST-1 assay result from the cells treated with CW-LPL and 1-Hz-30-% duty-cycle-PW-LPL with same 77 mJ/cm^2^ energy density. The metabolic activity was significantly enhanced when PW-LPL was applied in comparison with CW-LPL application. Alkaline phosphatase (ALP) activity was examined as an early marker for the osteogenic differentiation at elapsed time of 3 and 7 days from LPL treatments. Figure 2D shows that 1-Hz-30-% duty-cycle-PW-LPL increased the ALP activity significantly at elapsed time of 7 days from LPL treatment with energy density of both 77 mJ/cm^2^ and 2,310 mJ/cm^2^. The hDPSCs were similarly differentiated, even though the energy density was 3 times different. Together, cellular responses to PW-LPL did not depend upon the applied energy density but on duty cycle.

**Figure 3:**
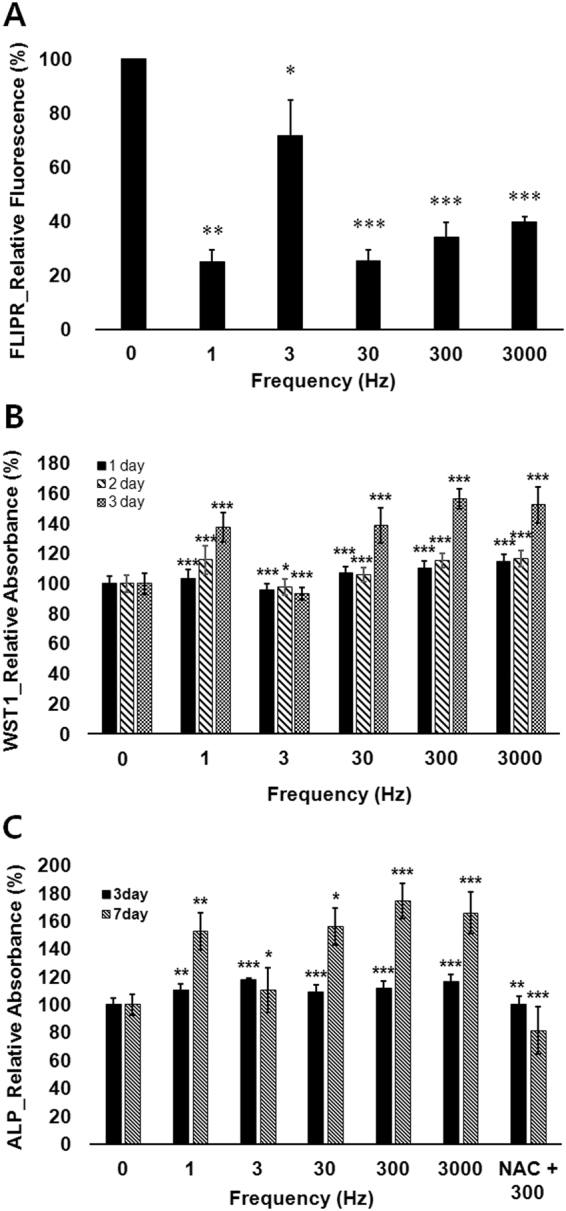
Effects of frequency of PW-LPL on hDPSC cytoplasmic membrane potential, metabolic activity, and ALP activity. (**A**) FLIPR fluorescence intensities of hDPSCs relative to control just after 77 mJ/cm^2^ PW-LPL in 1, 3, 30, 300, or 3000 Hz with same 30% duty cycle (n = 3 in each experiment and 3 replicates). (**B**) Metabolic activities of hDPSCs relative to control for 3 days after 77 mJ/cm^2^ PW-LPL in 1, 30, 300, or 3000 Hz with same 30% duty cycle (n = 10 in each experiment and 3 replicates). (**C**) ALP activity absorbance relative to control for 7 days after 77 mJ/cm^2^ PW-LPL in 1, 30, 300, or 3000 Hz with same 30% duty cycle. NAC was added for the case of 300 Hz PW-LPL (n = 10 in each experiment and 3 replicates).

In addition to the intensity and duty cycle, the frequency of pulse can be influential for biological consequences. To assess the effect of frequency of pulse on hDPSCs, we fixed the duty cycle 30% and the energy density 77 mJ/cm^2^ over all frequencies (0, 1, 3, 30, 300, 3000 Hz). CMP, cellular metabolic activity and ALP activity were assessed. Figure 3A shows that cells have hyperpolarized fashion in all frequencies right after LPL treatment. Figure 3B shows the WST-1 assay result at elapsed time of 24, 48, and 72 hours from PW-LPL treatment. Cellular metabolic activity was increased in most frequencies tested here. Figure 3C shows the analysis of ALP activity at elapsed time of 3 and 7 days from PW-LPL treatment with different frequencies. All cells that were subjected to the LPL revealed high ALP activity, which supports upregulated-osteogenic differentiation. With varying frequency of modulating LPL in a same dose, the metabolic activity of hDPSCs was enhanced most with 300 Hz PW-LPL.

Previous studies showed the relationship between the CMP and the progress of stem cell differentiation^34–37^. Authors found that the hyperpolarized CMP is related to quiescence of cells and the depolarized CMP is generally corresponding to proliferation and cell cycle progression. Cells in S-phase of mitosis have lower CMP difference (depolarized) and cells in cell cycle arrest have higher CMP difference (hyper-polarized). In case of differentiation, CMP increases as differentiation proceeds, since cells must coordinate their exit from the cell cycle with the initiation of their differentiation programs^36^. The relationship between CMP and differentiation was confirmed with chemical reagents which modulated CMP^34^. Our observation of spontaneous increase of CMP and the following increase of ALP activity indicates that molecules related to ion currents through membrane can be targets of LPL inducing cellular physiological responses including stem cell differentiation.

These experiments provide the optimal PW-LPL conditions for efficient hDPSC differentiation. hDPSC differentiation occurred more efficiently in PW stimuli than CW as previously reported with other cells^11,12^. 30-%-duty cycle was most efficient, and the frequencies between 1 and 3000 Hz with same energy density drove similar responses except 3 Hz. Exceptionally, 3 Hz showed little effects on all the measurements. The peculiar response to 3 Hz PW-LPL should be studied separately. The power up to 2.3 J/cm^2^ induced similar hDPSC responses. Therefore, for following experiments, we used PW-LPL with 30% duty cycle and 77 mJ/cm^2^ energy density in the range of 1–3000 Hz frequencies.

### Intracellular reactive oxygen species (ROS) and mitochondrial responses

One of the purposes in this study was to search for cellular factors to induce osteogenic differentiation. We estimated the qualitative changes of intracellular ROS after PW-LPL with addition of N-acetyl cysteine (NAC). Figure 3C showed that the addition of NAC during PW-LPL treatment reduced the increase of ALP activity. Since NAC is a well-known ROS scavenger, the enhanced ALP activity might be mediated by intracellular ROS^28^. Among various ROS, mitochondrial O_2_∙^−^ and cytoplasmic ROS levels were evaluated. Mitochondrial O_2_∙^−^ is generated as a byproduct of oxidative phosphorylation, and generally accepted as the major intracellular source of ROS. The cytoplasmic ROS includes OH∙, NO∙, or ROO∙, which can be produced from enzymes as well as from mitochondria.

The mitochondrial membrane potential (MMP) is an electrochemical gradient through the mitochondrial inner membrane which is controlled by respiratory complexes I, III, IV and anion channels and drives the synthesis of ATP. The instant increase of MMP and ATP was observed in various cells right after LPL irradiation via enhanced cytochrome c oxidase activity^30,31,37,38^. Cytochrom C oxidase is well known to absorb specifically NIR light in the range of 810–830 nm^39,40^. Karu *et al*. showed cytochrome C oxidase, the terminal enzyme of the mammalian respiratory chain, may play the main role in the initiation of LPL induced signal cascades^30^. MMP is delicately balanced by the concentration of ATP, ROS and Ca^2+^ ions^41^. In normal conditions, increase of MMP is strongly correlated with mitochondrial ATP and ROS generation, which are consistent with our observations.

**Figure 4:**
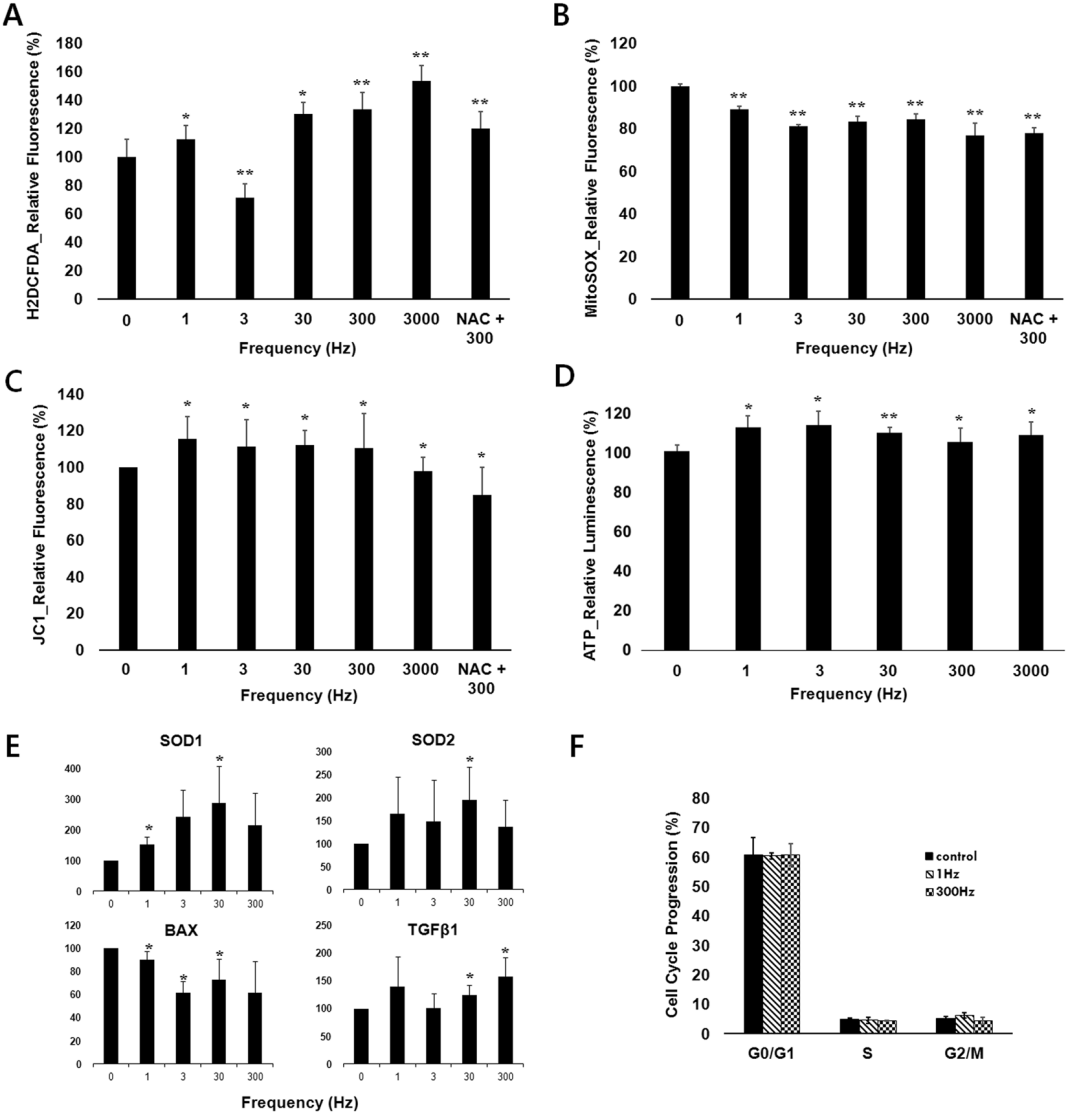
Effects of frequency of PW-LPL on intracellular ROS, mitochondrial activity, intracellular ATP of hDPSCs, changes in the nuclear transcriptional levels, and cell cycle cycles. LPL conditions were 1, 3, 30, 300, or 3000 Hz PW-LPL with same 30% duty cycle and 77 mJ/cm^2^ total energy density, and NAC was for the case of 300 Hz PW-LPL. Cells were stained by the following dyes; (**A**) H2DCFDA, (**B**) MitoSOX, (**C**) JC1, and (**D**) ATP-luciferase. All the values were expressed as relative intensities to control. Intracellular ROS, mitochondrial membrane potential, and intracellular ATP increased, while mitochondrial superoxide decreased. hDPSCs were treated by PW-LPL of 1, 3, 30, and 300 Hz PW-LPL with 30% duty cycle daily for 3 days (n = 3 in all each experiment and 3 replicates). (**E**) mRNA transcriptional levels for SOD1, SOD2, BAX, and TGF-β1. There was no statistics (n = 3 in each experiment and 3 replicates). (**F**) The population ratio in each cell cycle was not changed significantly. Increase of intracellular ROS affects the transcription of redox contolling genes, however it did not induce apoptosis or cell cycle arrest. There was no statistics (n = 3 in each experiment and 3 replicates).

Figure 4A shows that the formation of cytoplasmic ROS was promoted over all frequencies except for that of 3 Hz. On the contrary, Fig. 4B shows that the production of O_2_∙^−^ in mitochondria was considerably decreased compared with the control over all the frequencies. In order to verify whether the reduction in mitoSox was mediated by the reduction of mitochondrial activity, MMP was evaluated. Figure 4C shows the enhancement of MMP which is generally related to active mitochondrial states. Figure 4D shows the increased amount of the intracellular ATP, which is also related to high activity of mitochondria.

These results showed that PW-LPL enhanced the mitochondrial bioenergetics, reduced mitochondrial superoxide, but at the same time enhanced cytosolic ROS. The ROS are made either in the matrix or in the inter-membrane space of mitochondria and then diffused into cytosol through the mitochondrial membrane. It is known that blocking of respiratory components decreases MMP and increases ROS. The increase of both MMP and ROS, in our experiments, implies that PW-LPL irradiation regulates some other components which initiate a cascade of redox changes and modulation of biochemical reactions. PW-LPL treatment may affect the efflux of O_2_∙^−^ from mitochondrial inner membrane to cytoplasm. In previous reports, Aon *et al*. showed that a flash light could induce mitochondrial oscillation accompanying oscillating ROS formation, implicating the balance between ROS generation and their scavenging underlying the mitochondrial network^31^. Otherwise, PW-LPL may specifically activate mitochondrial complex III which enhanced ROS in cytosol easier than complex I which increases the MMP with generating ROS inside inner membrane of mitochondria^42^.

### Nucleus transcription factors

The increased cytoplasmic ROS has known to regulate nucleus transcription factors which determines cellular fates into proliferation, differentiation, or apoptosis, etc.^43^. The transcription factor nuclear factor kappa B (NF-kB) is one of the main transcription factors in redox signaling, which induce antioxidant enzymes. Superoxide dismutase (SOD) 1 and 2, superoxide scavengers, are both regulated mainly by NF-kB^44^. SOD1 is located in cytoplasm and SOD2 is located in mitochondria. Both are essential for the survival of aerobic organisms, and their mutations are related to various kinds of diseases. Figure 4E show that both SOD1 and SOD2 are upregulated over all the frequencies, which implies that cellular oxidative stress was increased and defense systems were initiated.

If the amount of ROS is too high, other transcription factors are activated to turn cell fate into cell cycle arrest or apoptosis. Tumor protein P53 is one of the important transcription factors inducing growth arrest in respond to oxidative stresses. Bcl-2-associated X protein (Bax) is one of the proteins transcribed by p53, which induces apoptosis. Figure 4E shows the transcription of Bax genes was reduced following three days of PW-LPL treatment, but transforming growth factor β1 (TGF- β1) was up-regulated. TGF- β1 is a multifunctional peptide whose dysregulation is known to result in apoptosis. In addition, Fig. 4F shows the population in each cell cycle after three days of LPL treatment. The populations in G_0_/G_1,_ S and G_2_/M were not significantly changed, which indicates that our PW-LPL treatment did not cause excessive levels of ROS to induce cell cycle arrest or apoptosis in hDPSCs^45^. Cells became 100% confluent after three days of culture regardless LPL treatment. Based on this result, the enhancement of WST-1 measurement may be ascribed to the enhanced metabolic activity of mitochondrial enzyme rather than cell proliferation.

Arany *et al*. showed that non-ionizing LPL treatment activate endogenous TGF-β1 through the increase of ROS^28^. TGF-β1 is known to be a master regulator of hDPSCs into dentinogenic differentiation. In their research, TGF- β1 signals were enhanced with the increase of LPL-treatment time, and the tendency was similar in ALP activities. Interestingly, our real time polymerase chain reaction data showed that the changes in TGF-β1 level according to the PW-LPL frequencies were similar to those in ALP activities. The transcription level was enhanced in most cases except when the frequency was 3 Hz. Though we could not reveal the underlying mechanisms for this frequency dependency, this correlation implies the strong relationship between TGF- β1 signaling and dentinogenic differentiation of hDPSCs.

### Dentinogenic differentiation

**Figure 5:**
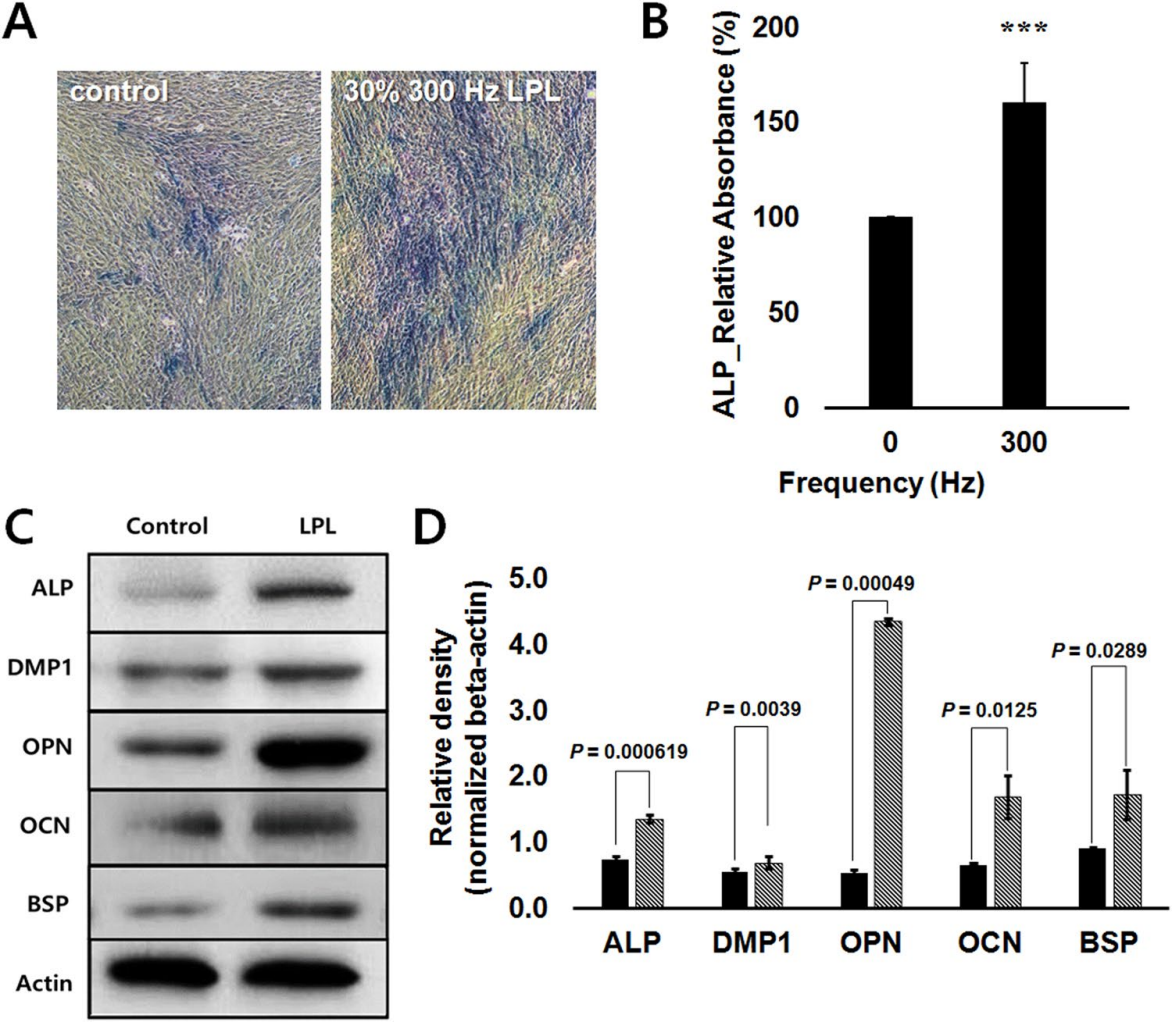
Dentinogenic differentiation of hDPSCs after 21 days of LPL daily treatment. 300 Hz PW-LPL with duty cycles 30% and 77 mJ/cm^2^ total energy density were applied. (**A**) Bright field images of hDPSCs stained with ALP staining kit at 21 days after 30%-3Hz-LPL daily irradiation. (**B**) ALP activity absorbance relative to control (n = 3 in each experiment and 3 replicates). (**C**) Western blots (W.B.) for ALP, DMP1, OPN, OCN, and BSP. (**D**) Bar graph showing mean density relative to beta-actin by analyzing Fig. 5C. The significance between controls and the LPL was all much lower than 0.05 (n = 3 in each experiment and 3 replicates).

Our measurements of changes in CMP, ALP activity, and metabolic enzyme activity support the possibility of commitment of hDPSCs into a dentinogenic lineage. To verify the induction of dentinogenic differentiation, morphological changes, ALP activity, and protein levels of ALP, dental matrix protein 1 (DMP1), osteopontin (OPN), osteocalcin (OCN), and bone sialoprotein (BSP) were estimated after 21 days of PW-LPL treatment with the frequency of 300 Hz. 300 Hz was chosen because cell’s spontaneous responses were significant at that frequency. Figure 5A show bright field images of hDPSCs stained with ALP activity assay kit after 21 days of daily PW-LPL irradiation. The stronger blue color implies the higher activity of ALP. This was confirmed quantitatively by using colorimetric analysis. Figure 5B shows that ALP activity became significantly higher at 21 days of 300-Hz-PW-LPL. Figure 5C shows the protein expression levels of dentinogenic related proteins^28,46^. The original gel images were added in supporting information. All proteins were upregulated, and the quantification showed statistical significance in Fig. 5D. These data confirm that LPL treatment promoted differentiation as well as commitment of hDPSC into a dentinogenic lineage.

In this study, we investigated several cellular responses to PW-LPL which are related to dentinogenic differentiation. Immediately after LPL treatment, CMP increased, MMP decreased, intracellular ROS increased, intracellular ATP increased, and mitochondrial ROS decreased. Three days after LPL treatment, transcription of SOD1, SOD2, TGF-β1 and ALP increased and that of BAX decreased. The long-term cellular differentiation should be ascribed to those initial cellular responses, and their relationships have been studied by many researchers. Sunderlacruz *et al*. showed that the inhibition of the inhibition of CMP reduced the osteogenic differentiation, and inversely increase of CMP enhanced the differentiation^34^. Pietila, *et al*. showed the strong correlations of MMP with ALP activity and together with Ca deposition^47^. Arany *et al*. showed that the level of ROS is strongly related to dentinogenic differentiation^28^. It is generally known that the low level of ROS contributes to maintain stem and progenitor cell’s stemness, while mild level of ROS promotes differentiation, proliferation, migration and survival of the stem cells and progenitor cells^41,48^.

Though our results have similar tendency to those previous works, we found that CMP and intracellular ROS have much higher correlation with ALP activity, a marker for dentinogenic differentiation, than the values related to mitochondrial activity. Mitochondria has been suggested as a main absorber of NIR light which responds immediately^30^. To induce whole cell responses such as proliferation and differentiation, the changes in mitochondria should be transferred to a cell nucleus. In case of PW-LPL with 3 Hz frequency, it induced similar mitochondrial responses but differential whole cell responses in comparison to other frequencies. For example, while most PW-LPL decreased mitochondrial superoxide and increased intracellular ROS, both mitochondrial and intracellular ROS were reduced with only 3Hz-LPL. 3Hz-LPL may activate the decomposition of ROS, inhibit the transfer of ROS through mitochondrial membrane, or modulate the lifetime of ROS itself. Considering the immediate change of CMP and MMP, the ion transfer seems definitively affected by PW-LPL. As suggested in previous studies, NIR light therapy may increases the intracellular ROS through transient mitochondrial activation, which may ultimately alter nuclear gene expression such as TGF-β1^28^. The pulsing of light may activate or disturb these processes, especially in the mitochondrial signaling to cytoplasm.

## Conclusions

In conclusion, we clearly showed that pulsing of LPL was more efficient in hDPSC differentiation than continuous wave irradiation. In our experiment, CW mode light did not induce significant changes in hDPSC states, which may be ascribed to the weak sub-mW level power of light we used. However, PW mode irradiation of the same power light induced significant changes in CMP and ALP activity. 30% duty cycle and 300–3000 Hz pulse frequencies showed the highest effects on hDPSC function. Though the mechanism is not clearly known, high production of intracellular ROS and the activation of TGF-β1 signaling pathway should be related to this pulsing mode enhanced hDPSC-dentinogenic differentiation *in vitro*. Our results let us put another step further to understand and utilize LPL therapy in dental medicine.

## Materials and Methods

### Fabrication of LPL system and application to cell culture plates

To make a uniform LPL emitting device, we assembled LEDs, a reflector, a light guide, and a diffuser as shown in Fig. 1. We used a reflector with diffuser that was used for TV manufacturing. The light intensity was measured using a photodiode (Model: S1227-66BQ) and the intensity variance was calculated by the intensities of three points marked with crosses (x) in Fig. 1C. We used LEDs as a light source whose wavelength is centered at 810 nm. The near infrared (NIR) light was confirmed using a spectrometer (Model: PMA-12, Japan). An operating device was designed and fabricated based on an 8-bit-microcontroller (UM_MC95FG308_V3.20_EN, Korea) and coded with C-language for adjusting the waveform, the duty cycle, and treatment time.

Light dose was checked before every experiment with a power meter (PM-USB-100, Thorlabs, USA). The assessed power density (irradiance, W/cm^2^) on a site of placing a plate in a continuous wave form was 268 μW/cm^2^ and it was reduced with the duty cycle. For example the power density became 30% when the duty cycle became 30%. The pulsed wave was in a square wave form as shown in Fig. 1E. For example, 1-Hz-square wave with 30% duty cycle means that LEDs were ‘on’ for 300 ms and ‘off’ for 700 ms, and 1-Hz-60-%-square wave is that LEDs were ‘on’ for 600 ms and ‘off’ for 400 ms. Duty cycles and frequencies could be easily controlled with keeping the same peak irradiance. Duty cycles of 0, 0.3, 3, 30, and 60% and the frequencies of 1, 3, 30, 300, 3000 Hz were used in this experiment. To make the irradiated light energy same (energy fluence 77 mJ/cm^2^) in both a continuous wave and pulsed waves, the irradiation time was controlled. For example, the irradiation time was 287 s for a continuous wave and 958 s for a pulsed wave. In an experiment to compare the effect of total applied energy, the application time was changed (Fig. 2B). In an experiment to determine the most effective duty cycle, we used the same irradiation time though the total energy was different (Fig. 2A). All the experimental conditions are summarized in a Table 1.

**Table 1:** The experimental conditions for this study.

Mode	PW 1 Hz				PW30%	CW	PW 30%				
	0.3%	3%	30%	60%	1 Hz		1 Hz	3 Hz	30 Hz	300 Hz	3000 Hz
Irradiance (μW/cm^2^)	0.8	8.0	80.4	160.8	80.4	268	80.4				
Time (s)	958	958	958	958	28731	287	958				
Fluence (mJ/cm^2^)	0.8	7.7	77	154	2310	77					

### Cell culture

Human molars were obtained from patients. Relevant informed consent was obtained from all participants. Patients consented the use of teeth for research purposes, and no information about patients was included in this article. This article does not contain information or images that could lead to identification of a study participant. The protocol was approved by the Institutional Review Board of Seoul National University Dental Hospital (Seoul, South Korea; IRB number 05004). All experiments were performed in accordance with relevant guidelines and regulations following previously confirmed protocols^49^. Briefly, the tooth specimens were dissected aseptically to disclose a pulp chamber and performed scraping tissues that contain pulp stem cells with a metal scraper. The separated tissues were incubated with 4 ml of 0.25% trypsin-EDTA (Life Technologies) at 37 °C for 30 min, and neutralized with 4 ml of a complete medium which contains α-MEM, 10% FBS and 1% penicillin (100 U/ml)-streptomycin (100 μg/ml) supplemented with 100 μM ascorbic acid (all from Gibco, Life Technologies). Then, solutions were pipetted vigorously and passed through a 40-μm cell strainer (Corning) to remove tissue debris. The obtained cells were cultured in a complete medium in a 37 °C incubator with 5% CO_2_, and the colonies were subcultured and expanded to obtain hDPSCs. The hDPSCs in the passage 3–5 were used in this experiment following previous studies. For dentinogenic differentiation of hDPSCs, 10 mM β-glycerophosphate, 0.05 mM L-ascorbic acid-2-phosphate and 100 nM dexamethasone were added. All the chemicals were purchased from Sigma-Aldrich. For LPL treatment, cells were seeded in a 4-well plate with a density of 2.0 × 10^4^ cells/well a day before treatment. For differentiation test (alkaline phosphatase assay and differentiation-related molecular assays) the differentiation medium was daily replaced before LPL treatment, and for the other tests the complete medium was daily replaced before LPL treatment. The morphology of cells was observed under inverted microscope (Eclipse Ti, Nikon, Japan).

### Measurement of cytoplasmic membrane potential

The cytoplasmic membrane potential (CMP) was assessed soon after LPL treatment by FLIPR (Molecular Devices, CA) according to the manufacturer’s protocol. Simply, cells were washed with DPBS one time and FLIPR working solution (FLIPR solution mixed with culture medium by halves) was added. After 30-minute of incubation at room temperature, cells were washed with DPBS, harvested with typsin-EDTA and resuspended in DPBS. The fluorescence of each cell was measured by flow cytometry (BDVerse, BD, Germany) and the mean value of each group was analyzed by the BD FACSuite^TM^ software. The relative intensity of each group to control was expressed as a percent value. The original flow cytometry data is applied in supporting information.

### Alkaline phosphatase (ALP) assay

hDPSCs were cultured to be confluence of over about 80% in culture wells. Then the culture media was replaced with differentiation media, and at the same time, LPL treatment was conducted every day for 3 days or 7 days before alkaline phosphatase (ALP) assay. In order to observe ALP expression in cells, we stained cells with SensoLyte® pNPP Alkaline Phosphatase Assay kit. Briefly, the prepared cells were gently washed twice with 1-X assay buffer. Then, lysis buffer (50 mM Tris HCL and 0.1% Triton X 100, pH 9.5, Sigma) was added in the cells and the cells were incubated for 10 min at 4 °C. After vibrating the cells for 60 sec, the cell supernatant was transferred into 96-well plate in an amount of 50 μl per well. pNPP substrate of 50 μl was added into each well and mixed the supernatant by gently shaking the plate for 30 sec. The cells were incubated for reaction at 37 °C for 60 min. The absorbance was assessed with a microplate reader (Synergy HT, Biotek) and expressed as a relative value to the control.

### WST- 1 assay

For WST-1 assay (CytoSelect™), cells were incubated in complete media with 10% WST-1 for 1 hr at 37 °C after 24, 48 and 72 hours of LPL treatment. The media was transferred to a new 96-well plate in amount of 100 μl per well and the absorbance was monitored at 450 nm through a plate reader (Tecan, USA).

### Adenine tri-phosphatase (ATP) assay

For ATP evaluation, the cells of 1.0 × 10^3^ cells/well were seeded in a 96-well white plate (3610, Corning). After 6 hours of seeding, the cells were treated by LPL and the media was washed with DPBS before adding CellTiter-Glo® Luminescent Cell Viability Assay (G7572, Promega). The plate was shaken for 5 minutes in medium strength, and the luminescence was measured by a plate reader (Synergy HT, Biotek). The luminescence of each group was expressed relatively to control as a percent value.

### Intracellular reactive oxygen species (ROS) and mitochondrial membrane potential (MMP, ∆ψ_m_)

After 5-minute of LPL treatment, cells were washed and the ROS probes were added. MitoSOX Red dye (M36008, ThermoFisher Scientific) for superoxide in mitochondria and H2DCFDA (D399, ThermoFisher Scientific) for intracellular ROS were used. MitoSOX was diluted to 5-μM in HBSS buffer, and cells were incubated in the solution for 10 min at 37 °C. After washing with DPBS and trypsinization, the fluorescence per a cell was measured by flow cytometry (BDVerse, BD, Germany). H2DCFDA was diluted to 10 μM in DPBS, and cells were incubated in the solution for 30-min at 37 °C. After washing three times with DPBS, cells were incubated for next 30 min, and the fluorescence per a cell was measured by flow cytometry. The relative intensity of each group to the control was expressed as a percent value.

Mitochondrial membrane potential (MMP) was detected by using MitoProbe^TM^ JC-1 assay kit (Thermo fisher Scientific). Cells were incubated in a complete media containing of JC-1 dye (2 μM) for 30 minutes and washed three times with DPBS. After trypsinization, the fluorescence per a cell was measured by flow cytometry. The relative intensity of each group to control was expressed as a percent value.

### Real time polymerase chain reaction

After 3 days of daily LPL irradiations in differentiation media, cells were harvested, lysed, and total RNAs were extracted using RNeasy Mini Kit (Qiagen). The total RNAs were converted to cDNAs using reverse transcriptase and random primers (cDNA synthesis kit, Toyobo) according to manufacturer’s protocol. The same amount of extracted total RNA taken from each sample was used in cDNA synthesis. The synthesized cDNAs were used in real time PCR using a CFX96^TM^ Real-Time System (BioRad). The relative gene expression was evaluated by the comparative cycle-threshold method. The relative amount of mRNA expression was normalized by that of RPL13α and expressed as a fold change to control. Primer sequences are as follows: RPL13α (F: 5′ CTATGACCAATAGGAAGAGCAACC, R: 5′ GCAGAGTATATGACCAGGTGGAA), SOD1 (F: 5′ GGCAAAGGTGGAAATGAAGA, R: 5′ GGGCCTCAGACTACATCCAA), SOD2 (F: 5′ GTTGGCCAAGGGAGATGTTA, R: 5′ TAGGGCTGAGGTTTGTCCAG), BAX (F: 5′ AACATGGAGCTGCAGAGGAT, R: 5′ CAGTTGAAGTTGCCGTCAGA), TGF-β1 (F: 5′ ACCTTGGGCACTGTTGAAGT, R: 5′ CTCTGGGCTTGTTTCCTCAC).

### Western blotting

After 21 days of daily LPL irradiations in differentiation media, the total proteins were isolated using a RIPA lysis buffer (Santa Cruz Biotechnology, Inc., Santa Cruz, CA, USA) supplemented with protease inhibitor mixture according to the manufacturer’s instruction. Cell lysates were centrifuged with 13,000 rpm at 4 °C, and the sediments were removed. The protein concentrations were determined using a BCA protein assay (Thermo Fisher Scientific, Inc, Rockford, IL, USA). Protein from the cell lysates was mixed with 5 × SDS-PAGE loading buffer and boiled for 10 min, prior to electrophoresis by 10% SDS-PAGE. Following transfer onto methanol-activated polyvinylidene difluoride membranes and blocking, the membranes were incubated overnight at 4 °C with polyclonal antibodies for alkaline phosphatase (ALP; Abcam, Cambridge, MA, USA), osteopontin (OPN; Merkmillipore, Billerica, MA, USA), dentin matrix protein 1 (DMP1; Arigo Biolaboratories Corp., Taiwan), ostocalcin (OCN), bone sialoprotein (BSP; Santa Cruz Biotechnology), monoclonal antibodies for beta actin (1:500, Santa Cruz Biotechnology, Inc., Santa Cruz, CA, USA). All antibodies were diluted with phosphate-buffered saline (PBS) solution containing 1% non-fat dry milk. Following three times washes in TBST (Tris-buffered saline, 0.1% Tween 20), the membranes were subsequently incubated for 1 hr with horseradish peroxidase-conjugated goat anti-rabbit antibody (Bio-Rad Laboratories, California, USA) or goat anti-mouse antibody (Bio-Rad Laboratories) diluted 1:5,000 in blocking buffer. The membranes were then washed three times with TBST, and signals were detected by pierce ECL system (Thermo Fisher Scientific, Inc.) and exposed to X-ray film. The quantitative densitometric value of each protein was normalized to ß-actin and displayed in histograms.

### Statistical analysis

All the experiments were repeated at least 3 times, and the data were expressed as the mean and standard deviation (SD). Statistical significance was evaluated using unpaired Student’s t-tests (two-tail, equal SD) with Microsoft Excel. Here, * indicates the *P*-value is under 0.05, ** under 0.01 and *** under 0.001. We denoted these stars when the value was significantly different from the control value, and *P* values were marked on the graph when the value was compared with other group.

## Electronic supplementary material

Supporting information
