# Supplementary material for: Effects of pulsing of light on the dentinogenesis of dental pulp stem cells in vitro
Source: Sci Rep. 2018 Feb 1;8:2057. doi: 10.1038/s41598-018-19395-x (PMC5795010; doi:10.1038/s41598-018-19395-x)
Supplement: Supplementary file 1 — Supporting information [file 41598_2018_19395_MOESM1_ESM.pdf]

## Supporting informations

# Effects of pulsing of light on the dentinogenesis of dental pulp stem cells *in vitro*

Hong Bae Kim[1†], Ku Youn Baik[2†], Hoon Seonwoo[3], Kyoung-Je Jang[1], Myung Chul Lee[1], Pill-Hoon Choung[4], Jong Hoon Chung[1*]

[1]Department of Biosystems & Biomaterials Science and Engineering, Seoul National University, Seoul, 08826, Republic of Korea

[2]Electrical and Biological Physics, Kwangwoon University, Seoul, 01897, Republic of Korea

[3]Department of Industrial Machinery Engineering, Sunchon National University, Jeollanam-do, 57922, Republic of Korea

[4]Department of Oral and Maxillofacial Surgery and Dental Research Institute, School of Dentistry, Seoul National University, Seoul, 03080, Republic of Korea

[*]Corresponding author e-mail: **jchung@snu.ac.kr**

†Both authors contribute equally to this work

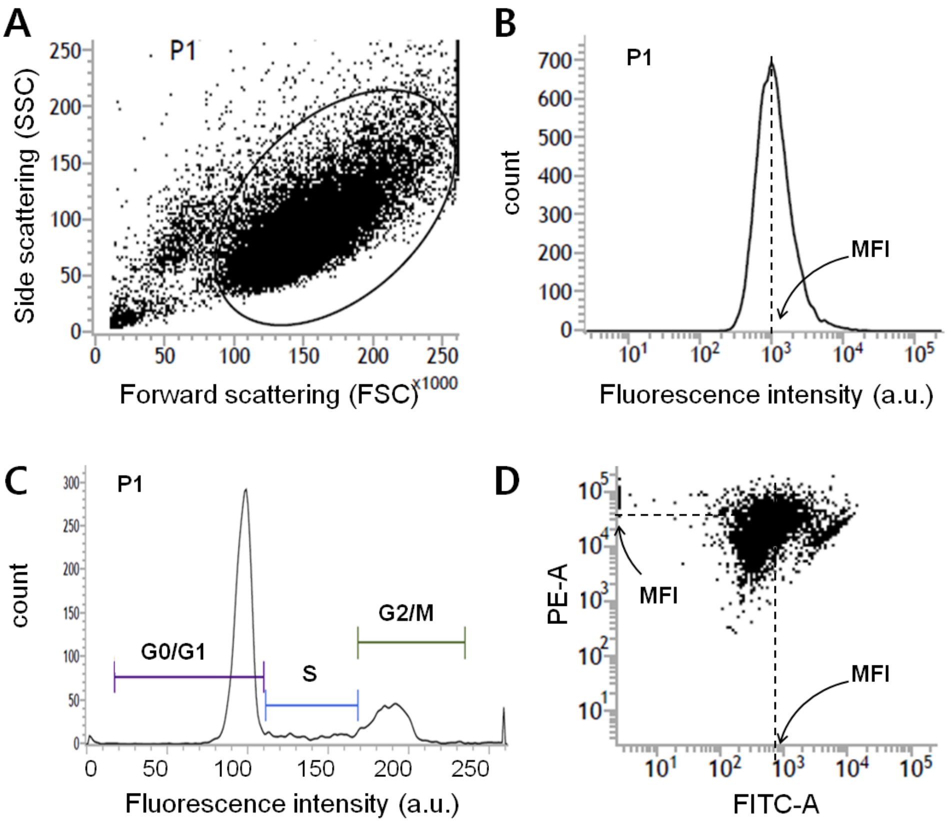


Figure S1. Typical graphs used to analyze the flow cytometry data. **A**. A typical 2D plot of forward (FSC) and side (SSC) scattering. Cells in certain ranges of FSC and SSC values were gated for further analysis. **B**. A typical graph of cell number distribution for one fluorescence measurement. The area integral of fluorescence intensity was used for measurement of the value per a cell. Mean fluorescence intensity (MFI) of each group was used for statistical analysis. **C**. A typical cell cycle graph, whose x-axis is the intensity of propidium iodide which is linearly increasing with DNA contents of a cell. It is divided to three parts as G0/G1, S, and G2/M states, and the population belong to each part is analyzed. **D**. A typical 2D graph of JC1 stained cells. The MFIs of FITC and PE were measured separately and the ratio of PE/FITC was used as a mitochondrial membrane potential.

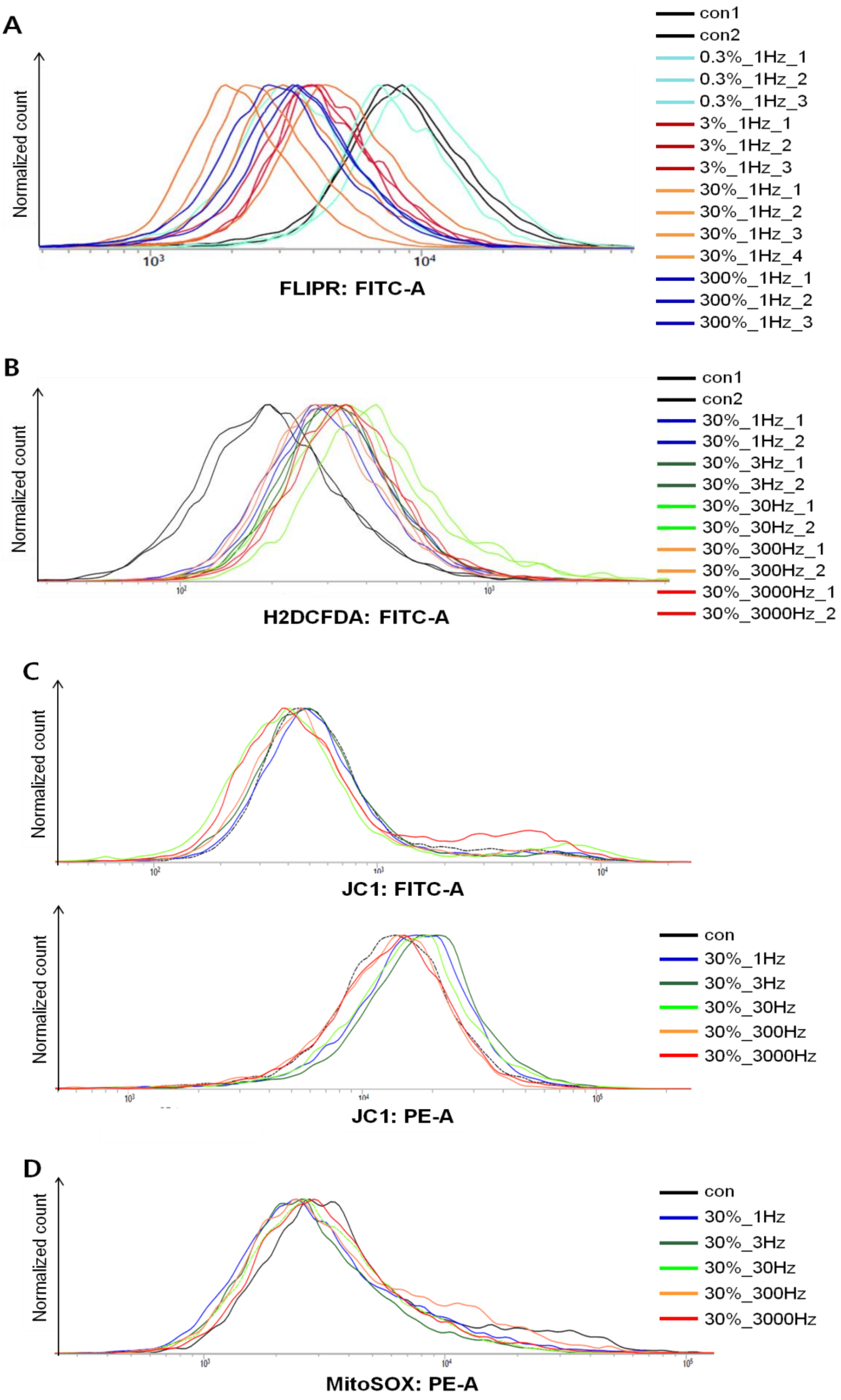


Figure S2. Typical data for measurement of cellular immediate responses to PW-LPL. Cells were stained with **A**. FLIPR, **B**. H2DCFDA, **C**. JC1, or **D**. MitoSOX.

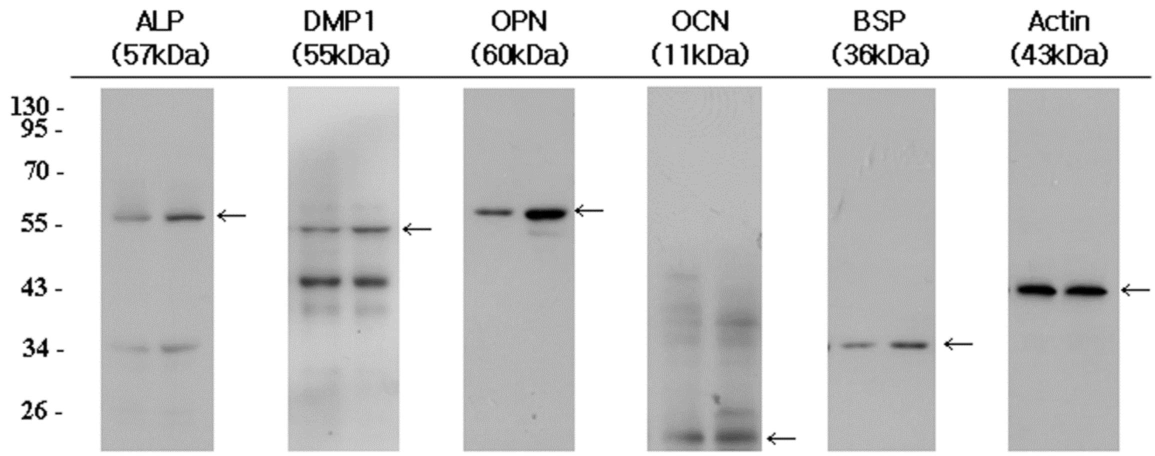


Figure S3. The original gel images for the protein expression of ALP, DMP1, OPN, OCN, BSP, Actin.
